# Supplementary material for: Adipose fin development and its relation to the evolutionary origins of median fins
Source: Sci Rep. 2019 Jan 24;9:512. doi: 10.1038/s41598-018-37040-5 (PMC6346007; doi:10.1038/s41598-018-37040-5)
Supplement: Supplementary file 1 — Supplementary Information [file 41598_2018_37040_MOESM1_ESM.pdf]

Adipose fin development and its relation to the evolutionary origins of median fins

Thomas A. Stewart[1]*, Melvin M. Bonilla[1], Robert K. Ho[1], and Melina E. Hale[1]

[1]Department of Organismal Biology and Anatomy
The University of Chicago
1027 E. 57th St, Chicago, IL 60637

***Correspondence to:** Thomas A. Stewart, Department of Organismal Biology and Anatomy, The University of Chicago, 1027 E. 57th St, Chicago, IL 60637; email: tomstewart@uchicago.edu

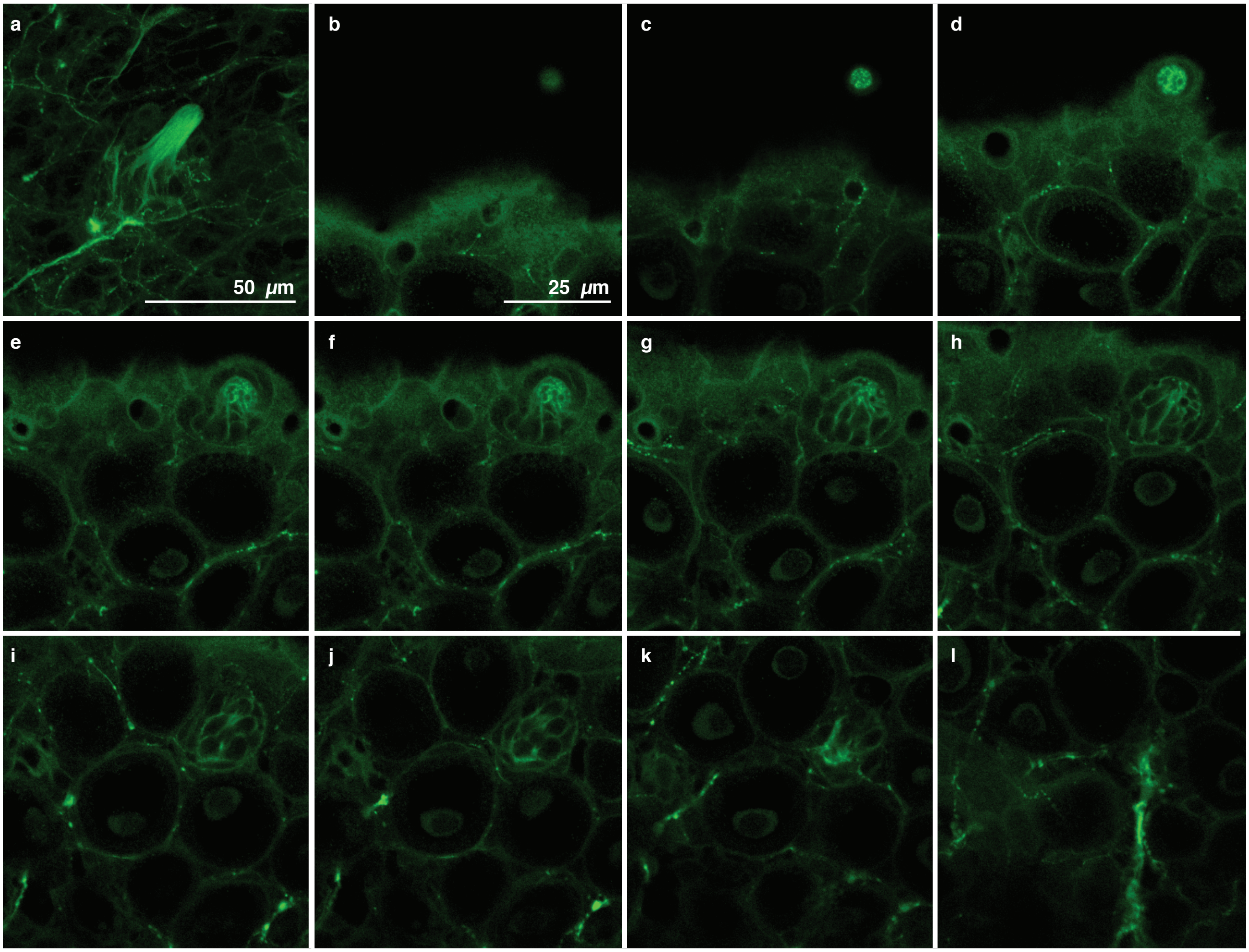

Supplementary Figure 1. Superficial neuromasts on the adipose fin spine and lateral scutes of *C. aeneus*. (a) Maximum-intensity projection of a superficial neuromast on the adipose fin spine. Panels (b-l) are of a neuromast on a lateral scute, which is oriented perpendicular to the image plane. Panels are from apex (b) to base (l). Depth between panels is 2.7 μm.

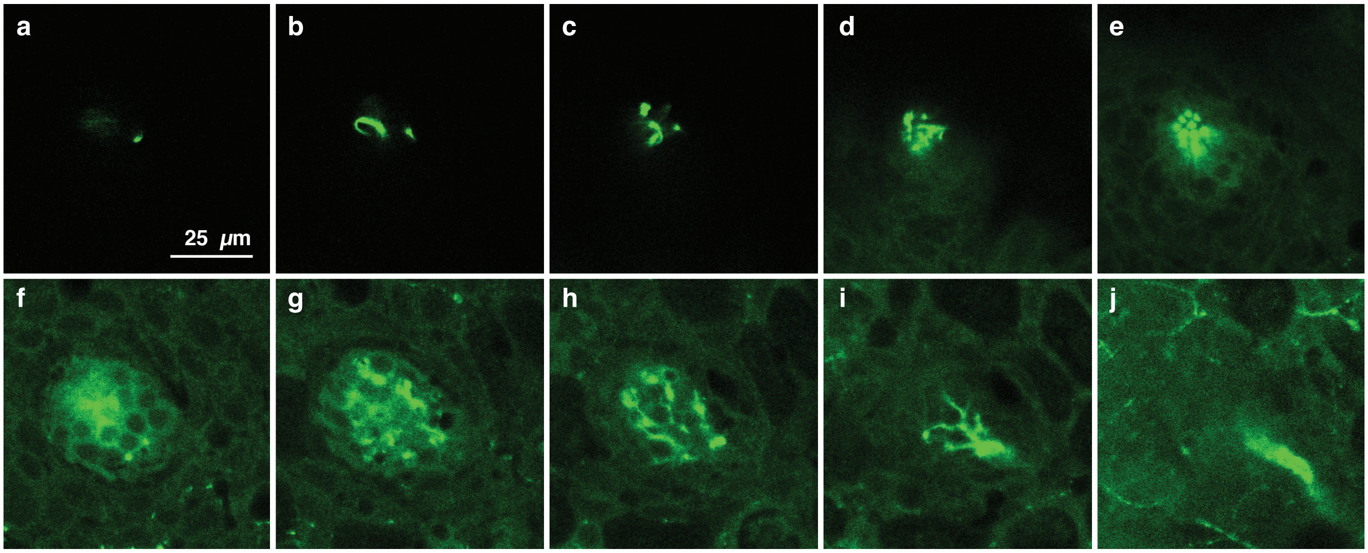

Supplementary Figure 2. Anatomy of a lateral line cell of *C. aeneus*. Panels (a-j) are of a lateral line cell positioned ventral to the adipose fin on the left side of the body; anterior is left. The lateral line cell is oriented perpendicular to the image plane, and panels are organized from apex (a) to base (j). The apex of the lateral line cell is characterized by a filamentous tip. Depth between panels is 5.5 μm.

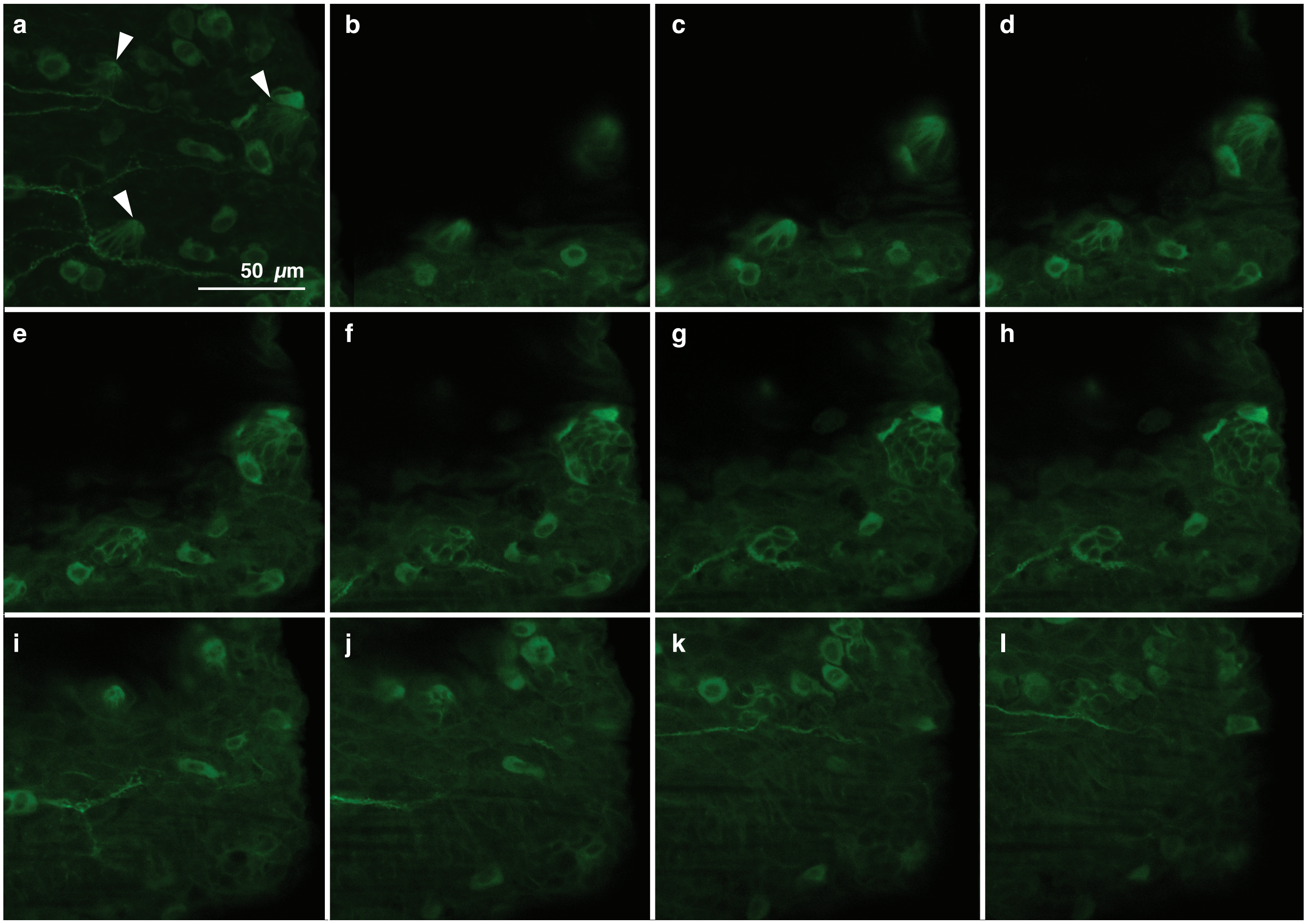

Supplementary Figure 3. Anatomy of a taste buds on the adipose fin of *C. aeneus*. (a) Maximum intensity projection showing three taste buds, indicated by arrowheads, and associated nerves. The taste buds are oriented perpendicular to the image plane, and panels are from apex (b) to base (l). Adipose fin afferents, which run parallel to actinotrichia in the membrane, terminate upon taste buds. Depth between panels is 3 μm.

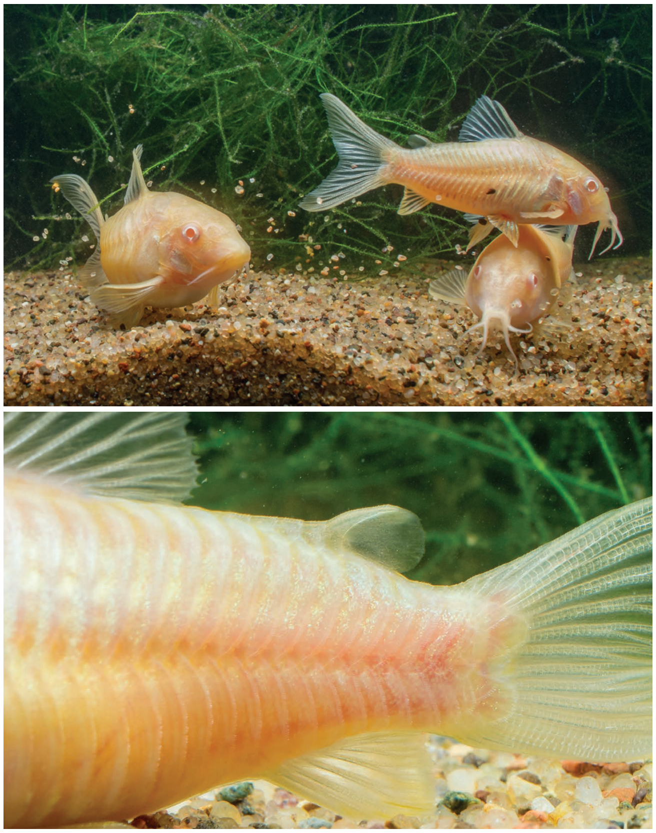

Supplementary Figure 4. Photographs of adult *C. aeneus* by Yen-Chyi Liu.

Suppl. Table 1. Data aggregated from the literature on adipose fin developmental diversity. Information on adipose fin development was found in one previous study of the fin's development[35], the taxonomic keys of larval fishes, and in developmental staging papers. The information is summarized in Fig. 1.

**order**	**family**	**genus**	**species**	**dev. mode**	**reference**
Siluriformes	Callichthyidae	*Corydoras*	*aeneus*	fold	this study, [72]
Siluriformes	Callichthyidae	*Corydoras*	*paleatus*	fold	[73]
Siluriformes	Callichthyidae	*Hoplosternum*	*littorale*	fold	[74]
Siluriformes	Ictaluridae	*Ictalurus*	*catus*	fold	[75]
Siluriformes	Ictaluridae	*Ictalurus*	*punctatus*	fold	[75]
Siluriformes	Ictaluridae	*Noturus*	*insignis*	fold	[75]
Characiformes	Alestidae	*Brycinus*	*longipinnis*	*de novo*	[35]
Characiformes	Alestidae	*Phenacogrammus*	*interruptus*	*de novo*	[35]
Characiformes	Characidae	*Astyanax*	*Mexicanus*	*de novo*	[76]
Characiformes	Characidae	*Hemigrammus*	*erythrozonus*	*de novo*	[35]
Characiformes	Characidae	*Hyphessobrycon*	*herbertaxelrodi*	*de novo*	[35]
Characiformes	Characidae	*Pironobrama*	*filigera*	*de novo*	[35]
Characiformes	Serrasalmidae	*Pygocentrus*	*nattereri*	*de novo*	[35]
Characiformes	Ctenoluciidae	*Ctenoluccius*	*hujeta*	*de novo*	[35]
Salmoniformes	Salmonidae	*Salmo*	*salmar*	fold	[77]
Salmoniformes	Salmonidae	*Salmo*	*trutta*	fold	[35]
Salmoniformes	Coregonidae	*Coregonus*	*maraena*	fold	[35]
Myctophiformes	Myctophidae	*Benthosema*	*glaciale*	fold	[78]
Myctophiformes	Myctophidae	*Ceratoscopelus*	*maderensis.*	fold	[78]
Myctophiformes	Myctophidae	*Electrona*	*risso*	fold	[78]
Myctophiformes	Myctophidae	*Diaphus*	*garmani*	fold	[79]
Myctophiformes	Myctophidae	*Benthosema*	*suborbitale*	fold	[80]
Myctophiformes	Myctophidae	*Centobranchus*	*nigroocellatus*	fold	[80]
Osmeriformes	Osmeridae	*Osmerus*	*eperlanus*	fold	[35]
